# Assessment of quality of life after upper extremity transplantation: Framework for patient-reported outcome scale domains

**DOI:** 10.3389/fpsyg.2022.989593

**Published:** 2023-01-24

**Authors:** Callie E. Tyner, Jerry Slotkin, Pamela A. Kisala, L. Scott Levin, Scott M. Tintle, David S. Tulsky

**Affiliations:** ^1^Center for Health Assessment Research and Translation, University of Delaware, Newark, DE, United States; ^2^Department of Orthopedic Surgery, Penn Medicine, Philadelphia, PA, United States; ^3^Department of Surgery, Division of Plastic Surgery, Penn Medicine, Philadelphia, PA, United States; ^4^Department of Orthopedic Surgery, Walter Reed National Military Medical Center, Bethesda, MD, United States; ^5^Department of Surgery, Uniformed Services University of the Health Sciences, Bethesda, MD, United States; ^6^Department of Physical Therapy, University of Delaware, Newark, DE, United States; ^7^Department of Psychological and Brain Sciences, University of Delaware, Newark, DE, United States

**Keywords:** quality of life, patient-reported outcomes, upper extremity, amputation, transplant

## Abstract

Upper extremity transplantation offers the promise of restored function and regained quality of life (QOL) for individuals who have sustained hand or arm amputation. However, a major challenge for this procedure becoming an accessible treatment option for patients is the lack of standard measures to document benefits to QOL. Patient-reported outcomes (PRO) measures are well-suited for this kind of intervention, where the perspective of the patient is central to defining treatment success. To date, qualitative work with experts, clinicians, and patients has been used to identify the most important domains of QOL for PRO item development. Specifically, our group’s qualitative work has identified several domains of QOL that are unique to individuals who have received upper extremity transplants, which are distinct from topics covered by existing PRO measures. These include emotional and social aspects of upper extremity transplant, such as Expectations and Perceived Outcomes, Integration and Assimilation of Transplant, Fitting in, and Post-Surgical Challenges and Complications. The broad topic of Satisfaction with Transplant was subdivided into three subtopics: Function, Sensation, and Aesthetics. Satisfaction with Sensation was also identified as a unique domain not evaluated by existing PRO measures. This report operationalizes these eight QOL domains by presenting scoping definitions. This manuscript describes the work that has been completed for domain characterization as an early step toward developing standardized PRO measures to evaluate these important outcomes specific to upper extremity transplantation.

## Introduction

Upper extremity (UE) limb injury and limb loss have been found to affect multiple areas of functioning including emotional well-being, social functioning (Paterson and Burke, 1995; Desmond, 2007; Saradjian et al., 2008; Gallagher et al., 2011; Østlie et al., 2011; Desteli et al., 2014), and physical functioning including activities of daily living, secondary medical conditions, and pain (Madhok and Bhopal, 1992; Davidson et al., 2010; Postema et al., 2012; Passero, 2014). UE limb loss drastically changes multiple aspects of one’s quality of life (QOL), thus producing a need for QOL-improving rehabilitative treatments. UE transplant *via* vascularized composite allotransplantation (VCA) offers one such QOL-improving treatment option for those with hand and arm amputation (Elliott et al., 2014; Tintle et al., 2014).

Upper extremity transplantation has been shown to be a surgically and medically feasible treatment option for UE limb loss and is being offered at several surgical centers across the globe (Hollenbeck et al., 2009; Kaufman et al., 2009; Kubiak et al., 2019). However, the QOL outcomes from UE transplant have not yet been systematically or comprehensively documented (Kumnig et al., 2014; Bound Alberti et al., 2022). UE functioning is the primary outcome currently tracked for UE transplant recipients (Petruzzo et al., 2010; Tintle et al., 2014; Shores et al., 2017), for example using the Disabilities of the Arm, Shoulder, and Hand (DASH) measure (Beaton et al., 2001). However, by focusing only on UE functioning, this outcome assessment approach provides a limited picture of the success of UE VCA. While UE functional outcomes are important, they may not be sufficient to justify what is ostensibly a QOL intervention. This is particularly true given the current availability of advanced functional prosthetic arms and hands (Kubiak et al., 2019), which may confer function without necessarily addressing other aspects salient to QOL. Therefore, the full QOL impacts of this intervention remain to be documented objectively. Small samples and single-case studies using limited psychosocial outcomes have been reported (Singh et al., 2015; Salminger et al., 2016). However, the best metrics for evaluating QOL comprehensively for this population remain unknown.

With support from the U.S. Department of Defense’s Defense Health Program Congressionally Directed Medical Research Programs, our research team has embarked on a program of study to develop a standardized and comprehensive approach for assessing the QOL outcomes of UE transplant that can be applied across treatment centers to allow for more uniform tracking of outcomes. We are using the methodology for patient-reported outcomes (PRO) measure development outlined and demonstrated by the Patient Reported Outcomes Measurement Information System (PROMIS®) initiative funded by the National Institutes of Health Common Fund (PROMIS, 2013) and the Quality of Life in Neurological Disorders (Neuro-QoL™) measurement system funded by the National Institute of Neurologic Disorders and Stroke (National Institutes of Health, n.d., Fries et al., 2005, Cella et al., 2006, 2007, 2011, Northwestern University, 2021). PROs are lacking for UE transplant, although this format for outcomes assessment is well suited for a QOL-improving intervention like VCA, given that the perspective of the patient is central to defining treatment success. Members of our research team have previously used these methods for developing condition-specific PRO measurement systems for spinal cord injury (Tulsky et al., 2011, 2015b,c; Tulsky and Kisala, 2015) and traumatic brain injury (Tulsky et al., 2016; Tulsky and Kisala, 2019), and we are currently developing item banks for limb injury and amputation (Tyner et al., 2018).

The initial steps in this PRO development process are to obtain substantive stakeholder feedback to guide development of such a system and to review existing measures for content (Bjorner and Ware, 1998; Bjorner et al., 2007; De Walt et al., 2007; Kisala and Tulsky, 2010; PROMIS, 2013). Thus far, our team has documented the domains of QOL impacted by UE transplant by soliciting stakeholder input (Tulsky et al., 2023) using a grounded theory-based qualitative approach (Glaser and Strauss, 1967; Strauss and Corbin, 1998; Kisala and Tulsky, 2010). The findings from this work are described briefly below. Our qualitative work has outlined domain areas specific to stakeholders of UE transplantation and we have used this information to determine where new PROs are needed. The present report describes these newly identified domains and the approach for drafting new PRO items.

## Materials and methods

### Qualitative stakeholder focus groups and individual interviews

Stakeholder feedback was collected from UE transplant experts and clinicians (i.e., surgeons, nurses, mental health professionals, and physical and occupational therapists) through a series of focus groups, and from patients who have received UE transplant *via* individual telephone interviews. The full methodology and findings have been reported in a separate manuscript (Tulsky et al., 2023) but will be described here briefly.

Three focus groups were conducted at the 2018 meeting of the American Society for Reconstructive Transplantation, and 10 focus groups were held across five UE transplant centers in the United States in 2019–2020. In total, 59 clinicians and other UE transplant experts along with five UE transplant recipients provided input on the most important domains of health-related QOL (HRQOL) for UE transplant. Trained data collectors and doctoral-level co-investigators conducted all sessions using open-ended semi-structured discussion guides to lead the groups or interviews. Sessions were audio-recorded and transcribed. All participants provided informed consent and data collection was approved by the University of Delaware Institutional Review Board.

This stakeholder input was analyzed qualitatively using a grounded-theory-based (Glaser and Strauss, 1967; Strauss and Corbin, 1998) approach that our team has used in several prior studies to identify important HRQOL domains in other clinical populations (Kisala and Tulsky, 2010; Slavin et al., 2010; Carlozzi et al., 2011; Tulsky et al., 2011, 2015b, 2016). Open, axial, and selective coding were employed to determine the important HRQOL themes (MacQueen et al., 1998; Morgan et al., 1998; Braun and Clarke, 2006; Guest et al., 2012).

The results demonstrated that there are numerous domains relevant to HRQOL after UE transplantation that can be measured by existing PRO systems that cover topics salient to many other clinical populations (e.g., fine motor functioning, anxiety, and pain interference). However, several domains of HRQOL that are unique to UE transplantation were identified as well. This highlighted the need for new item development to ensure HRQOL assessments are comprehensive. The framework defined in this manuscript provides the roadmap for how to draft new PRO items and demonstrates how the qualitative results helped define these new HRQOL domains.

### Identifying gaps in HRQOL measurement for UE transplant and defining domains

New HRQOL content domains important to UE transplant recipients were identified in response to the qualitative input from stakeholders (Tulsky et al., 2023). The content coverage of each domain was designed to be directly responsive to the comments from stakeholders. Quotes from the interviews or focus groups were used as source material for new item text. To guide the future development of items, domain definitions were articulated and refined for each of the new domain areas.

## Results

**Figure 1:**
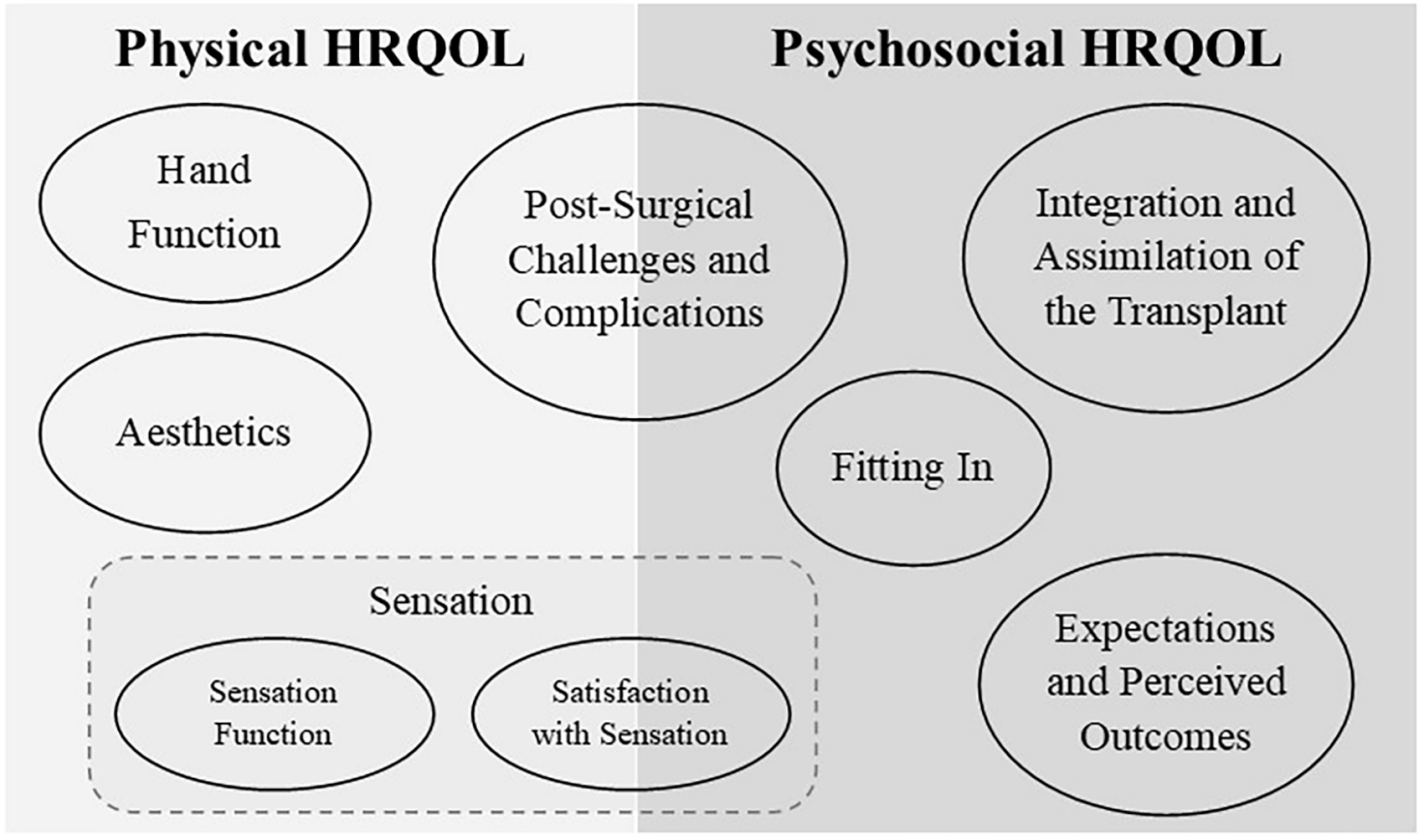
The eight new HRQOL content domains are presented above—represented by ovals—grouped into the overarching area of physical and psychosocial HRQOL under which they fall. In the area of physical HRQOL, three domains relevant to satisfaction with transplant were identified: Hand Function, Aesthetics, and Sensation. Sensation was further subdivided by separating Sensation Function from Satisfaction with Sensation. One domain includes aspects of both physical and psychosocial HRQOL: Post-Surgical Challenges and Complications, which includes physical and emotional reactions post-surgically. Psychosocial HRQOL included reflection on past perspectives in light of the completed transplant (Expectations and Perceived Outcomes). The internal reactions to stigmatization or feelings of self-acceptance (Integration and Assimilation of the Transplant) were separated from the external experience of stigmatization/acceptance within the social context (Fitting In).

Eight new HRQOL content domains were developed based on stakeholder input. The subject, span, and relevant subtopics of each domain were defined. See Table 1 for a brief summary of these eight HRQOL content domains. Figure 1 contains a visual representation of how the domains are related across psychosocial and physical HRQOL. Each section below begins with a summary of the stakeholder input and exemplar quotes from the stakeholders are provided. Information on the subtopics considered for new PRO item drafting are described and examples of items are provided. These draft items will go through several stages of review and refinement before they can be considered ready for use; the draft items are presented here to demonstrate the process of PRO item development and are not intended to be adopted as PRO items for UE transplant HRQOL until the item development process is complete.

**Table 1:** New domains to evaluate HRQOL after UE transplant.

Domain	Content coverage
Expectations and Perceived Outcomes	Satisfaction with results of transplant and overall outcomes as well as accuracy of expectations in retrospect.
Post-Surgical Challenges and Complications	Burdens of post-transplant treatment and therapies; effects on health and personal life.
Integration and Assimilation of the Transplant	Acceptance and identification of the transplant as one’s own; feelings of being complete or having something restored.
Fitting In	Comfort in social interactions where other people may view or touch the transplant(s).
Satisfaction with Hand Function	Comfort, confidence, and satisfaction with the functional abilities of the transplant(s) in various daily activities.
Satisfaction with Hand Aesthetics	Satisfaction with physical appearance of the transplant.
Hand Function: Sensation	Ability to perceive sensations with the transplant.
Satisfaction with Sensation	Satisfaction with sensory abilities of the transplant.

### Expectations and perceived outcomes

Stakeholders described how it was difficult for UE transplant recipients and their families to truly know what the transplantation and recovery process was going to be like, despite extensive attempts by providers to inform them. Preparing UE transplant recipients for the requirements of recovery—and tempering expectations—was described as a major part of the pre-surgical evaluation process at all sites where experts were interviewed.

*“Well, I expected to not [feel good] and they told me that, you would not feel good for a while. They were very honest.”* –UE transplant recipient

*“I don't know you really ever completely prepare for that…. I had always been healthy.”* –UE transplant recipient

*“We aren't selecting patients for hand transplant who walk away saying, ‘I want to play the piano and I expect to play the piano.’ So, one of the selection criteria is that they have low expectations and high motivation.… Someone whose need is to look completely normal in society and function as a concert pianist is not going to get a hand transplant from our group.”* –UE transplant expert stakeholder

*“We definitely assess what their expectations are pre-transplant. And…if there's a big mismatch between their pre-transplant expectation and the reality of their outcome then we will see depression. But it's really important to assess whether or not they have realistic expectations and whether they understand what the potential adverse outcomes could be.”* –UE transplant expert stakeholder

To assess how well recipients’ pre-surgical expectations were met, we developed the Expectations and Perceived Outcomes domain. Stakeholder comments coded in this domain included emotional topics, such as feeling happy with results, second-guessing choices, and feeling regret or surprise about the outcome. Experiences and expectations of surgery, rehabilitation, medical side effects, and immediate recovery were also discussed. These emotional and practical topics will be incorporated into items in this domain; for example, *I felt prepared for the risks to my health after hand transplant*.

### Integration and assimilation of the transplant

Stakeholders described the process and importance of integrating the UE transplant into one’s physical and mental bodily schema. This included how the recipients experienced the process of accepting the UE transplant as their own limb and also how the UE transplant changed how they felt about their bodies.

*“So many of those patients, they've tried prosthetics and ultimately, they always see the prosthetic as a foreign object; they're never able to integrate this in their overall body image. This was always a foreign device. Whereas after the hand transplant they referred to the transplant as their own hand, so I think that's something that explains a little bit what it means, this wholeness part. Where with the prosthesis, although they have regained function where you can do things, they never felt whole; they always felt as an amputee. Whereas after transplant they have hands and they feel whole again by using those hands.”* –UE transplant expert stakeholder

*“It makes me feel whole*.” –UE transplant recipient

The Integration and Assimilation of the Transplant domain was conceptualized to assess feelings about how well the UE transplant has been integrated and/or assimilated into the recipient’s life, including somatosensory feelings of bodily integrity (e.g., proprioception and kinesthetic) as well as corresponding psychological experiences, sometimes referred to as a feeling of “wholeness” or being complete. For example, an item like, *My hand transplant makes me feel more complete*, could be representative of this domain. Stakeholder comments coded in this domain included references to acceptance of the limb, such as to what extent the new UE feels like their own. These topics and other salient emotional experiences were targeted for content coverage, such as degree of comfort (or discomfort) with the transplanted limb, feeling like oneself again, and feeling like something lost has been regained.

### Fitting in

Stakeholders discussed a number of important social experiences that are unique for UE transplant recipients, both prior to and after transplantation. Descriptions of the experience prior to the transplant were primarily about difficulties fitting in as an amputee, whereas after UE transplant, the comments were both positive and negatively valenced. In many cases recipients felt they were better able to fit in due to the transplanted limbs, although some concerns remained about being noticed as having post-transplant limb differences (for example, mismatches in size or skin tone). Salient emotions included fears of being judged negatively in social settings and relevant behaviors included avoidance of social contact due to concerns about not fitting in.

*“After getting the hands, I felt like [I] could blend in more.”* –UE transplant recipient

*“…[I]f by doing the transplant they can go out in the world and they're not immediately identified as an amputee; … it's not the first thing that people notice about them.”* –UE transplant expert stakeholders

The Fitting In domain was developed to evaluate the recipients’ feelings of how well they fit in socially after UE transplant. Stakeholder comments coded in this domain included references to feelings of social comfort or discomfort, feeling judged by others, and potential for embarrassment about the physical appearance or functioning of the transplanted limb. Several comments also referenced the importance of confidence interacting in social settings where the transplanted limb may be observed—for example, decisions about wearing long sleeves or short sleeves and concerns about others staring at their limbs. This was incorporated into an exemplar drafted item, *I feel self-conscious about people seeing my hands*. This domain also captures feelings about being socially accepted and feeling a sense of “normality” with regard to not standing out as different in some way when perceived by others. Topics targeted for content coverage included both positive and negative feelings about fitting in socially, and referenced different social settings, such as being in public or around friends and family.

### Post-surgical challenges and complications

Stakeholders described numerous challenges after transplantation, including adverse effects on health from immunosuppressive treatment and requirements for extensive rehabilitation care. There was also discussion of the occurrence of negative emotional outcomes from these new, potentially burdensome experiences. This content domain is multifaceted, covering a wide range of potential post-surgical challenges and complications, spanning aspects of both physical and psychosocial HRQOL (see Figure 1).

*“You end up living at the hospital essentially for a while. So, you leave your life and you come. What I taught patients is rehabilitation becomes your full-time job from here on out. Or at least, other outside interests that you have, they have to take a back seat if you want to do this. And this has to be what you do every day seven days a week. And it’s going to be this way for a year or maybe this way for two years.”* –UE transplant expert stakeholder

*“I do know that the side effects of the steroid that was given to me in such heavy doses caused me to gain a lot of weight.”* –UE transplant recipient

*“After about 5 years of having the hands, the [medication] really started to damage my kidneys.”* –UE transplant recipient

The Post-Surgical Challenges and Complications domain was conceptualized as encompassing common post-surgical challenges or problems that may have developed as a result of the transplant as well as associated ongoing physical and emotional burdens of transplantation. Stakeholder comments covered a wide range of topics, including burdens of keeping up with treatment, taking medications, regular checkups, required lifestyle changes, and hand therapy (appointments and at-home exercises). Some of these burdens were described as potentially lifelong. Discussions also described the emotional aspects of complications, including concern about medication side effects, long-term health impacts (e.g., cancer and diabetes), lifespan reduction, rejection episodes, and life-threatening or severe complications. Of note, many of these challenges and complications are faced by individuals who undergo solid organ transplant. Potential items such as, *“My treatment limits my leisure activities”* and *“I feel bothered by medication side effects,”* exemplify this domain. The experiences of recipients with any post-transplant challenges or complications, and the experiences of burden and emotional sequelae from these experiences, were judged to be most pertinent to inform item content coverage in this domain.

### Satisfaction with hand function

Stakeholders described that, depending on the recipient, different levels of functioning may be experienced as satisfactory. Some felt that very little UE functioning was a positive outcome, whereas other recipients were seen to have higher requirements for the level of functioning needed to feel satisfied. Discussions of UE transplant function included the movement, strength, and flexibility of the UE as well as feelings of comfort, confidence, and satisfaction with the UE transplant functionality.

*“My hand functions just as well as a normal hand, to be honest. I can fully grasp, make a fist… you forget that you are an amputee a lot of times.”* –UE transplant recipient

*“I'm very happy even if my hands do not move like normal hands.”* –UE transplant recipient

“*But then there are other patients who have completely different goals, and for them it's really to work out, to do pull-ups, whatnot*.” –UE transplant expert stakeholder

The Satisfaction with Hand Function domain was designed to capture satisfaction with the use and functionality of the transplant, considering the entire UE. Topics covered include general satisfaction and satisfaction with specific functions and uses (e.g., range of motion). Stakeholder comments coded in this domain included both positive and negative statements about the UE transplant functionality as well as descriptions of different activities that could or could not be done with the UE transplant. These different types of functionality and descriptions of emotional reactions to the functional outcomes are important aspects that inform the drafting of items in this domain—for example, *I feel frustrated with how my transplant functions*.

### Satisfaction with hand aesthetics

Stakeholders shared that the aesthetic aspects of the UE transplant were important for recipients’ evaluation of their overall outcome to a greater or lesser degree depending on the individual’s goals in seeking a transplant. For some, the aesthetic aspects were paramount and the procedure was tantamount to aesthetic surgery, while for others, the appearance of the transplant was a secondary or tertiary goal. Stakeholders also explained that the aesthetic aspects of the transplants were expected to change over time, as follow-up procedures (e.g., debulking) could be done to improve aesthetics.

*“It has been always important for me to have new real hands and not plastic or silicone hands.”* –UE transplant recipient

*“They try to match on skin color as well as [donor] sex.. but …there's often a big size discrepancy in the arms …because what's left of [the recipient’s] native arm is often very shrunken and small, and then you're transplanting … a normal size forearm.”* –UE transplant expert stakeholder clinician

To assess recipients’ satisfaction with the external appearance of the transplant, we designed the Satisfaction with Hand Aesthetics domain. Specific subtopics covered in the stakeholder discussions included skin tone of the transplant, size of the transplant, fingernail appearance, forearm bulk, scar appearance, and body hair color. Each item would cover an aspect of only one of these subtopics—for example; *I am satisfied with the skin tone of my transplant*. These subtopics and the need to evaluate the recipient’s feelings of satisfaction or dissatisfaction with the aesthetic qualities of the UE transplant were considered to be important considerations when drafting items for this domain.

### Hand function: Sensation and satisfaction with sensation

Stakeholders discussed sensation as a major outcome of interest that motivated many recipients to pursue UE transplant. Sensation is one of the functions that cannot be replicated by traditional prosthetics. Sensation has numerous important impacts on daily functioning as well as emotional and social functioning.

Stakeholders described the sensory skills that were missed for UE amputees, and the process of regaining sensation after transplantation. Various sensory skills were discussed as functional abilities, but also for the more personal meaning inherent in these sensory experiences. Sensation was discussed as a socially relevant sense and was closely tied to the desire for improved social functioning after the transplant.

*“Sensation though is so important. And I can’t reinforce that enough as it relates to relationships with those that you love. Your spouse and your children, especially for those that have young children. Hooks don’t have any value with young children, and electric hands don’t have value with children.”* –UE transplant expert stakeholder

*“I can feel what I touch, I can feel if it is hot or if it is cold, if it is soft, or if it is itchy or anything, and … that is something that is very important for me, and it goes with the fact that I can like touch somebody. So, for example, my boyfriend, I can… put my hand on him and I can touch him or feel him or touch his hair or things like that… that really matters for me currently.”* –UE transplant recipient

In response to these stakeholder comments, two HRQOL content domains were developed on the topic of sensation. First, the Hand Function: Sensation domain was designed to evaluate recipients’ ability to perceive a variety of sensations in the transplanted limb/hand. These included, for example, light pressure, touch, textures, temperature, and pain. The second domain developed was Satisfaction with Sensation. This domain was designed to assess recipients’ satisfaction with their ability to perceive sensation with the transplant, including social touch.

These two domains were conceptualized as discrete because stakeholders acknowledged that recipients’ degree of satisfaction may not correlate directly with the amount of sensory function they have in the UE transplant. Stakeholders described how acquiring even minimal amounts of protective sensation was experienced as a benefit over prosthetic devices. Hence, Hand Function: Sensation focuses on practical aspects of sensory skills and behaviors as shown in an exemplar item like: *My sense of touch in my hand(s) is good.* Satisfaction with Sensation focuses more on satisfaction and mental/emotional implications of regaining sensory functioning and can be depicted in an item such as: *My hand(s) help(s) me feel closer with people when I touch them.*

## Discussion

To understand the HRQOL effects of what is considered to be a QOL-enhancing a procedure, it is critical to systematically assess post-transplant HRQOL from the patient’s own perspective. The first steps to implementing routine PRO assessment in a given population are to identify the most important domains of HRQOL to assess, and then to develop PRO items to measure each of these domains. Many of the factors important to understanding the physical and psychosocial HRQOL outcomes of UE transplant are shared with other rehabilitation populations, such as fine motor functioning and ability to conduct self-care activities, pain interference, and emotional difficulties, such as depression, anxiety, and psychological trauma (Cella et al., 2012; Gershon et al., 2012; Kisala et al., 2015a,c; Tulsky et al., 2015a). Additionally, several unique areas relevant to HRQOL after UE transplantation were identified in our recent qualitative work with stakeholders (Tulsky et al., 2023).

After transplant, it is known that recipients face new challenges and risks of complications, and there are opportunities for hindsight and possible regrets, all of which have the potential for condition-specific psychosocial outcomes. These are areas where we determined that new PRO content was necessary, and so we are working to develop the domains of Expectations and Perceived Outcomes and Post-Surgical Challenges and Complications, the latter of which includes aspects of both physical and psychosocial HRQOL.

Psychosocial outcomes such as reduced participation and regaining independence are domains where existing PRO measures can be applied (Cella et al., 2012; Gershon et al., 2012; Heinemann et al., 2015, 2020; Victorson et al., 2015; Kisala et al., 2020). Likewise, measures of depression, anxiety, and traumatization are relevant for UE transplant recipients just as they are for individuals in other rehabilitation populations who have experienced life-altering disabilities (Kisala et al., 2015a,c; Tulsky et al., 2015b). However, the domains of stigmatization and self-acceptance, topics that have been identify as relevant for many other rehabilitation populations (Cella et al., 2012; Gershon et al., 2012; Kalpakjian et al., 2015; Kisala et al., 2015b) are experienced in a particular way by UE transplant recipients. These patients are faced with a unique opportunity to integrate the transplanted limb into their identity (i.e., assimilation), as well as an opportunity to reintegrate socially as a person with ostensibly intact limbs. The concepts of feeling “whole” and “normal”—terms that can evoke negative stereotypes and reflect the type of ableist language typically avoided in rehabilitation research—were repeatedly broached by stakeholders in our qualitative work. Use of these terms in this way implies that if the loss of a limb results in feeling like “something is missing,” a transplanted limb is indeed an opportunity to both figuratively and literally feel restored or “whole.” These sensitive topics are critical, therefore, for inclusion in assessment of HRQOL outcomes following UE transplant. To respond to this need, we have chosen to develop outcomes domains in the psychosocial HRQOL area of Integration and Assimilation of the Transplant and Fitting In.

From a medical/surgical perspective, satisfaction with the transplant is a broad and vital area for assessment. Based on our stakeholder input and experience with PRO development, we chose to divide the topic of physical HRQOL after UE transplant into three areas: Function, Sensation, and Aesthetics. Functional *abilities* of the transplanted limb can be evaluated similarly to any surgical population, considering, for example, range of motion, grip strength, pain, and the various activities of daily living that require manual motor function and dexterity to complete. Thus, we believe that assessment of functional ability is best left to existing measures, such as SCI-FI Fine Motor, SCI-FI Self-Care, PROMIS Upper Extremity (Jette et al., 2012; Tulsky et al., 2012; Kaat et al., 2019), or Neuro-QoL Upper Extremity-Fine Motor (Cella et al., 2012; Gershon et al., 2012).

In contrast, *satisfaction* with hand functioning as experienced by UE transplant recipients appears to merit a new HRQOL content domain, as difficulties and frustrations with the responsivity and ease of movement of the transplanted limbs/hands are distinct for this population, where capabilities improve gradually with treatment and nerve regrowth—or sometimes not at all. Likewise, the challenges with sensation and aesthetic satisfaction are also unique to UE transplant. Although there are other clinical groups (e.g., spinal cord injury) where UE sensation can be impacted by injury, and the process of developing sensory-motor control for advanced prosthetic limb users has some similarities (Graczyk et al., 2019; Sensinger and Dosen, 2020), the experience of regaining sensory capabilities as donor nerves become reinnervated is distinct in limb transplant. There are similarities for UE transplant recipients to the benefits experienced from targeted muscle reinnervation (TMR) in terms of reduced phantom limb pain and neuroma-related pain (Morgan et al., 2016; Tintle et al., 2016; Dumanian et al., 2019), and similarities with both invasive (e.g., targeted sensory reinnervation) and non-invasive (e.g., armband-based stimulators) technologies for restoring sensory input with prosthetic or bionic devices (Bensmaia et al., 2020), although ideally the outcome of the VCA transplant will go further than any one of these approaches and restore more natural motor control and sensation to the injured limb. Furthermore, the aesthetic and cosmetic aspects are of central concern for many patients (e.g., concerns about mismatched skin tone, body hair color/texture, and musculature/size/bulk of transplanted limb). These issues are layered upon the body image concerns experienced by UE amputees and involve the singular experience of aesthetically integrating donor tissue in a highly visible body location (whereas most solid-organ transplants occur with internal/non-visible tissue). Hence, we have chosen to develop outcomes measures in the physical HRQOL area, including Hand Function, Aesthetics, and Sensation (both Function and Satisfaction with Sensation).

The work in which we are engaged is designed to use stakeholder feedback to identify the appropriate domains for HRQOL assessment and to make the feedback actionable by developing items to measure these important stakeholder-identified areas. The definitions and theoretical structure described throughout this manuscript are critical to the development of items that can measure these areas of function. This marks one of the initial efforts to systematically develop new scales that focus directly on issues of critical importance to those who have undergone UE transplant, but which are absent from existing HRQOL measurement systems.

## Conclusion

Standardized, routine, and comprehensive evaluations of UE transplant outcomes are necessary to provide evidence to evaluate this treatment as part of the standard of care for UE injury or limb loss. Based on industry standards for PRO assessments of HRQOL and our completed qualitative research, we recommend that HRQOL assessment for this population includes both existing measures—those that are applicable to many rehabilitation populations, such as measures of depression, anxiety, and pain—as well as measures that are unique to the experience of UE transplant, covering emotional, social, and physical functioning. The eight newly developed PRO domains described herein were designed for this purpose. Future work is needed to finalize the development of new items in these domains and to ensure content validity.

## Data availability statement

The datasets presented in this article are not readily available because a data use agreement must be signed prior to release. Requests to access the datasets should be directed to DT, dtulsky@udel.edu.

## Ethics statement

The studies involving human participants were reviewed and approved by University of Delaware Institutional Review Board. The patients/participants provided their written informed consent to participate in this study.

## Author contributions

All authors contributed to data collection. CT, PK, and DT managed the qualitative analysis. CT, JS, and DT wrote new item domain scoping definitions. CT wrote the first draft of the manuscript. All authors contributed to the article and approved the submitted version.

## Funding

This work was supported by grant numbers W81XWH-18-2-0066, W81XWH-18-2-0067, and W81XWH-18-2-0068 from the U.S. Department of Defense, Defense Health Program, Congressionally Directed Medical Research Programs, Reconstructive Transplant Research Program, Qualitative Research Award.

## Conflict of interest

The authors declare that the research was conducted in the absence of any commercial or financial relationships that could be construed as a potential conflict of interest.

## Publisher’s note

All claims expressed in this article are solely those of the authors and do not necessarily represent those of their affiliated organizations, or those of the publisher, the editors and the reviewers. Any product that may be evaluated in this article, or claim that may be made by its manufacturer, is not guaranteed or endorsed by the publisher.

## Abbreviations

DASH: Disabilities of the Arm, Shoulder, and Hand
HRQOL: Health-related quality of life
Neuro-QoL™: Quality of Life in Neurological Disorders
PRO: Patient-reported outcome
PROMIS®: Patient Reported Outcomes Measurement Information System
QOL: Quality of life
TMR: Targeted muscle reinnervation
UE: Upper extremity
VCA: Vascularized composite allotransplantation

## References

[ref1] Beaton D. E. Katz J. N. Fossel A. G. Wright J. G. Tarasuk V. Bombardier C. (2001). Measuring the whole or the parts? Validity, reliability, and responsiveness of the disabilities of the arm, shoulder and hand outcome measure in different regions of the upper extremity. J. Hand Ther. 14, 128–142. doi: 10.1016/S0894-1130(01)80043-0 11382253

[ref2] Bensmaia S. J. Tyler D. J. Micera S. (2020). Restoration of sensory information via bionic hands. Nat. Biomed. Eng. doi: 10.1038/s41551-020-00630-8 PMC10233657 33230305

[ref3] Bjorner J. B. Chang C. H. Thissen D. Reeve B. B. (2007). Developing tailored instruments: item banking and computerized adaptive assessment. Qual. Life Res. 16, 95–108. doi: 10.1007/s11136-007-9168-6, PMID: 17530450

[ref4] Bjorner J. B. Ware J. (1998). Using modern psychometric methods to measure health outcomes. Med. Outcomes Trust Monitor. 3, 11–16

[ref5] Bound Alberti F. Ridley M. Herrington E. Benedict J. L. Hall S. (2022). What we still don't know about vascularized composite allotransplantation (VCA) outcomes and quality of life measurements. Transplant. Rev. 36:100708. doi: 10.1016/j.trre.2022.100708, PMID: 35644045

[ref6] Braun V. Clarke V. (2006). Using thematic analysis in psychology. Qual. Res. Psychol. 3, 77–101. doi: 10.1191/1478088706qp063oa

[ref7] Carlozzi N. E. Tulsky D. S. Kisala P. A. (2011). Traumatic brain injury patient-reported outcome measure: identification of health-related quality-of-life issues relevant to individuals with traumatic brain injury. Arch. Phys. Med. Rehabil. 92, S52–S60. doi: 10.1016/j.apmr.2010.12.046, PMID: 21958923

[ref8] Cella D. Lai J. S. Nowinski C. J. Victorson D. Peterman A. Miller D. (2012). Neuro-QOL: Brief Measures of Health-Related Quality of Life for Clinical Research in Neurology. Neurology 78, 1860–1867. doi: 10.1212/WNL.0b013e318258f744, PMID: 22573626 PMC3369516

[ref9] Cella D. Nowinski C. Peterman A. Victorson D. Miller D. Lai J. S. (2011). The neurology quality-of-life measurement initiative. Arch. Phys. Med. Rehabil. 92, S28–S36. doi: 10.1016/j.apmr.2011.01.025, PMID: 21958920 PMC3193028

[ref10] Cella D. Moy C. S. Victorson D. Nowinski C. Peterman A. Miller D. M. (2006). The Neuro-QOL Project: Using multiple methods to develop a HRQOL Measurement Platform to be Used in Clinical Research Across Neurological Conditions. Symposium presentation at the 13th Annual Conference of the International Society for Quality of Life Research. Published abstract. Qual. Life Res. 15:14–15. doi: 10.1007/s11136-007-9211-7

[ref11] Cella D. Yount S. Rothrock N. Gershon R. Cook K. Reeve B. (2007). The Patient-Reported Outcomes Measurement Information System (PROMIS): progress of an NIH roadmap cooperative group during its first two years. Med. Care 45, S3–S11. doi: 10.1097/01.mlr.0000258615.42478.55, PMID: 17443116 PMC2829758

[ref12] Davidson J. H. Khor K. E. Jones L. E. (2010). A cross-sectional study of post-amputation pain in upper and lower limb amputees, experience of a tertiary referral amputee clinic. Disabil. Rehabil. 32, 1855–1862. doi: 10.3109/09638281003734441, PMID: 20345252

[ref13] De Walt D. A. Rothrock N. Yount S. Stone A. A. Group PC (2007). Evaluation of item candidates: the PROMIS qualitative item review. Med. Care 45, S12–S21. doi: 10.1097/01.mlr.0000254567.79743.e2 17443114 PMC2810630

[ref14] Desmond D. M. (2007). Coping, affective distress, and psychosocial adjustment among people with traumatic upper limb amputations. J. Psychosom. Res. 62, 15–21. doi: 10.1016/j.jpsychores.2006.07.027, PMID: 17188116

[ref15] Desteli E. E. Imren Y. Erdogan M. Sarisoy G. Cosgun S. (2014). Comparison of upper limb amputees and lower limb amputees: a psychosocial perspective. Eur. J. Trauma Emerg. Surg. 40, 735–739. doi: 10.1007/s00068-014-0418-3, PMID: 26814792

[ref16] Dumanian G. A. Potter B. K. Mioton L. M. Ko J. H. Cheesborough J. E. Souza J. M. (2019). Targeted muscle reinnervation treats neuroma and phantom pain in major limb amputees: a randomized clinical trial. Ann. Surg. 270, 238–246. doi: 10.1097/SLA.0000000000003088, PMID: 30371518

[ref17] Elliott R. M. Tintle S. M. Levin L. S. (2014). Upper extremity transplantation: current concepts and challenges in an emerging field. Curr. Rev. Musculoskelet. Med. 7, 83–88. doi: 10.1007/s12178-013-9191-x, PMID: 24241894 PMC4094126

[ref18] Fries J. F. Bruce B. Cella D. (2005). The promise of PROMIS: using item response theory to improve assessment of patient-reported outcomes. Clin. Exp. Rheumatol. 23, S53–S57. 16273785

[ref19] Gallagher P. O'Donovan M. A. Doyle A. Desmond D. (2011). Environmental barriers, activity limitations and participation restrictions experienced by people with major limb amputation. Prosthetics Orthot. Int. 35, 278–284. doi: 10.1177/0309364611407108, PMID: 21937573

[ref20] Gershon R. C. Lai J. S. Bode R. Choi S. Moy C. Bleck T. (2012). Neuro-QOL: quality of life item banks for adults with neurological disorders: item development and calibrations based upon clinical and general population testing. Qual. Life Res. 21, 475–486. doi: 10.1007/s11136-011-9958-8, PMID: 21874314 PMC3889669

[ref21] Glaser B. G. Strauss A. L. (1967). The Discovery of Grounded Theory: Strategies for Qualitative Research. New Brunswick, NJ: Aldine Transaction.

[ref22] Graczyk E. L. Gill A. Tyler D. J. Resnik L. J. (2019). The benefits of sensation on the experience of a hand: a qualitative case series. PLoS One 14:e0211469. doi: 10.1371/journal.pone.0211469, PMID: 30703163 PMC6355013

[ref23] Guest G MacQueen K. M Namey E. E. (2012). Applied Thematic Analysis. Los Angeles: SAGE Publications, Inc.

[ref24] Heinemann A. W. Kisala P. A. Boulton A. J. Sherer M. Sander A. M. Chiaravalloti N. (2020). Development and Calibration of the TBI-QOL Ability to Participate in Social Roles and Activities and TBI-QOL Satisfaction With Social Roles and Activities Item Banks and Short Forms. Arch. Phys. Med. Rehabil. 101, 20–32. doi: 10.1016/j.apmr.2019.07.015, PMID: 31473208

[ref25] Heinemann A. W. Kisala P. A. Hahn E. A. Tulsky D. S. (2015). Development and psychometric characteristics of the SCI-QOL Ability to Participate and Satisfaction With Social Roles and Activities Item Banks and Short Forms. J. Spinal Cord Med. 38, 397–408. doi: 10.1179/2045772315Y.0000000028, PMID: 26010974 PMC4445030

[ref26] Hollenbeck S. T. Erdmann D. Levin L. S. (2009). Current indications for hand and face allotransplantation. Transplant. Proc. 41, 495–498. doi: 10.1016/j.transproceed.2009.01.065, PMID: 19328911

[ref27] Jette A. M. Tulsky D. S. Ni P. Kisala P. A. Slavin M. D. Dijkers M. P. (2012). Development and initial evaluation of the spinal cord injury-functional index. Arch. Phys. Med. Rehabil. 93, 1733–1750. doi: 10.1016/j.apmr.2012.05.008, PMID: 22609635

[ref28] Kaat A. J. Buckenmaier C. T. 3rd Cook K. F. Rothrock N. E. Schalet B. D. Gershon R. C. (2019). The expansion and validation of a new upper extremity item bank for the Patient-Reported Outcomes Measurement Information System (PROMIS). J. Patient Report. Outcomes 3:69. doi: 10.1186/s41687-019-0158-6, PMID: 31773413 PMC6879697

[ref29] Kalpakjian C. Z. Tate D. G. Kisala P. A. Tulsky D. S. (2015). Measuring self-esteem after spinal cord injury: development, validation and psychometric characteristics of the SCI-QOL Self-Esteem Item Bank and Short Form. J. Spinal Cord Med. 38, 377–385. doi: 10.1179/2045772315Y.0000000014, PMID: 26010972 PMC4445028

[ref30] Kaufman C. L. Blair B. Murphy E. Breidenbach W. B. (2009). A new option for amputees: transplantation of the hand. J. Rehabil. Res. Dev. 46, 395–404. doi: 10.1682/JRRD.2008.08.0108 19675991

[ref31] Kisala P. A. Tulsky D. S. (2010). Opportunities for CAT applications in medical rehabilitation: development of targeted item banks. J. Appl. Meas. 11, 315–330. 20847478 PMC6445378

[ref32] Kisala P. A. Tulsky D. S. Boulton A. J. Heinemann A. W. Victorson D. Sherer M. (2020). Development and psychometric characteristics of the TBI-QOL Independence item Bank and Short Form and the TBI-QOL Asking for Help Scale. Arch. Phys. Med. Rehabil. 101, 33–42. doi: 10.1016/j.apmr.2019.08.469, PMID: 31473207

[ref33] Kisala P. A. Tulsky D. S. Kalpakjian C. Z. Heinemann A. W. Pohlig R. T. Carle A. (2015a). Measuring anxiety after Spinal Cord Injury: Development and Psychometric Characteristics of the SCI-QOL Anxiety Item Bank and Linkage with GAD-7. J. Spinal Cord Med. 38, 315–325. doi: 10.1179/2045772315Y.0000000029, PMID: 26010966 PMC4445022

[ref34] Kisala P. A. Tulsky D. S. Pace N. Victorson D. Choi S. W. Heinemann A. W. (2015b). Measuring stigma after Spinal Cord Injury: Development and Psychometric Characteristics of the SCI-QOL Stigma Item Bank and Short Form. J. Spinal Cord Med. 38, 386–396. doi: 10.1179/1079026815Z.000000000410, PMID: 26010973 PMC4445029

[ref35] Kisala P. A. Victorson D. Pace N. Heinemann A. W. Choi S. W. Tulsky D. S. (2015c). Measuring psychological trauma after Spinal Cord Injury: Development and Psychometric Characteristics of the SCI-QOL Psychological Trauma Item Bank and Short Form. J. Spinal Cord Med. 38, 326–334. doi: 10.1179/2045772315Y.0000000022, PMID: 26010967 PMC4445023

[ref36] Kubiak C. A. Etra J. W. Brandacher G. Kemp S. W. P. Kung T. A. Lee W. P. A. (2019). Prosthetic rehabilitation and vascularized composite Allotransplantation following upper limb loss. Plast. Reconstr. Surg. 143, 1688–1701. doi: 10.1097/PRS.0000000000005638, PMID: 31136485

[ref37] Kumnig M. Jowsey S. G. Moreno E. Brandacher G. Azari K. Rumpold G. (2014). An overview of psychosocial assessment procedures in reconstructive hand transplantation. Transpl. Int. 27, 417–427. doi: 10.1111/tri.12220, PMID: 24164333

[ref38] MacQueen K. M. McLellan E. Kay K. Milstein B. (1998). Codebook development for team-based qualitative analysis. Cult. Anthropol. Methods. 10, 31–36. doi: 10.1177/1525822X980100020301

[ref39] Madhok R. Bhopal R. S. (1992). Coping with an upper limb fracture? A study of the elderly. Public Health 106, 19–28. doi: 10.1016/S0033-3506(05)80325-7, PMID: 1603913

[ref40] Morgan D. L. Krueger R. A. King J. A. (1998). Focus Group Kit. Thousand Oaks, CA: SAGE Publications

[ref41] Morgan E. N. Potter B. K. Souza J. M. Tintle S. M. Nanos G. P. III (2016). Targeted muscle reinnervation for transradial amputation: description of operative technique. Tech. Hand Up. Extrem. Surg. 20, 166–171. doi: 10.1097/BTH.0000000000000141, PMID: 27824734

[ref42] National Institutes of Health (n.d.). PROMIS: Clinical Outcomes Assessment. Available at: https://commonfund.nih.gov/promis/index

[ref43] Northwestern University (2021). Health Measures. Available at: http://www.healthmeasures.net/

[ref44] Østlie K. Magnus P. Skjeldal O. H. Garfelt B. Tambs K. (2011). Mental health and satisfaction with life among upper limb amputees: a Norwegian population-based survey comparing adult acquired major upper limb amputees with a control group. Disabil. Rehabil. 33, 1594–1607. doi: 10.3109/09638288.2010.540293, PMID: 21166612

[ref45] Passero T. (2014). Devising the prosthetic prescription and typical examples. Phys. Med. Rehabil. Clin. N. Am. 25, 117–132. doi: 10.1016/j.pmr.2013.09.009, PMID: 24287243

[ref46] Paterson M. C. Burke F. D. (1995). Psychosocial consequences of upper limb injury. J. Hand Surg. (Br.) 20, 776–781. doi: 10.1016/S0266-7681(95)80046-8, PMID: 8770740

[ref47] Petruzzo P. Lanzetta M. Dubernard J. M. Landin L. Cavadas P. Margreiter R. (2010). The international registry on hand and composite tissue transplantation. Transplantation 90, 1590–1594. doi: 10.1097/TP.0b013e3181ff1472, PMID: 21052038

[ref48] Postema S. G. van der Sluis C. K. Waldenlov K. Norling Hermansson L. M. (2012). Body structures and physical complaints in upper limb reduction deficiency: a 24-year follow-up study. PLoS One 7:e49727. doi: 10.1371/journal.pone.0049727, PMID: 23226218 PMC3511484

[ref49] PROMIS (2013). Instrument development and validation—Scientific standards (v2.0). Available at: https://www.fda.gov/media/137976/download

[ref50] Salminger S. Sturma A. Roche A. D. Hruby L. A. Paternostro-Sluga T. Kumnig M. (2016). Functional and psychosocial outcomes of hand transplantation compared with prosthetic fitting in below-elbow amputees: a multicenter cohort study. PLoS One 11:e0162507. doi: 10.1371/journal.pone.0162507, PMID: 27589057 PMC5010226

[ref51] Saradjian A. Thompson A. R. Datta D. (2008). The experience of men using an upper limb prosthesis following amputation: positive coping and minimizing feeling different. Disabil. Rehabil. 30, 871–883. doi: 10.1080/09638280701427386, PMID: 17852212

[ref52] Sensinger J. W. Dosen S. (2020). A review of sensory feedback in upper-limb prostheses from the perspective of human motor control. Front. Neurosci. 14:345. doi: 10.3389/fnins.2020.00345, PMID: 32655344 PMC7324654

[ref53] Shores J. T. Malek V. Lee W. P. A. Brandacher G. (2017). Outcomes after hand and upper extremity transplantation. J Mater. Sci. Mater. 28. doi: 10.1007/s10856-017-5880-0 28361279

[ref54] Singh M. Oser M. Zinser J. Sisk G. Carty M. J. Sampson C. (2015). Psychosocial outcomes after bilateral hand transplantation. Plast. Reconstr. Surg. Glob. Open 3:e533. doi: 10.1097/GOX.0000000000000520, PMID: 26579339 PMC4634170

[ref55] Slavin M. D. Kisala P. A. Jette A. M. Tulsky D. S. (2010). Developing a contemporary functional outcome measure for spinal cord injury research. Spinal Cord 48, 262–267. doi: 10.1038/sc.2009.131, PMID: 19841635

[ref56] Strauss A. L. Corbin J. (1998). Basics of Qualitative Research: Techniques and Procedures for Developing Grounded Theory 2nd Edn. Thousand Oaks, CA: SAGE Publications.

[ref57] Tintle S. M. LeBrun C. Ficke J. R. Potter B. K. (2016). What is new in trauma-related amputations. J. Orthop. Trauma 30, S16–S20. doi: 10.1097/BOT.0000000000000668, PMID: 27661421

[ref58] Tintle S. M. Potter B. K. Elliott R. M. Levin L. S. (2014). Hand transplantation. JBJS Rev. 2:1. doi: 10.2106/JBJS.RVW.M.00063 27490811

[ref59] Tulsky D. S. Jette A. M. Kisala P. A. Kalpakjian C. Dijkers M. P. Whiteneck G. (2012). Spinal cord injury-functional index: item banks to measure physical functioning in individuals with spinal cord injury. Arch. Phys. Med. Rehabil. 93, 1722–1732. doi: 10.1016/j.apmr.2012.05.007, PMID: 22609299 PMC3910090

[ref60] Tulsky D. S. Kisala P. A. (2015). The Spinal Cord Injury--Quality of Life (SCI-QOL) Measurement System: development, psychometrics, and item bank calibration. J. Spinal Cord Med. 38, 251–256. doi: 10.1179/2045772315Y.0000000035, PMID: 26010961 PMC4445017

[ref61] Tulsky D. S. Kisala P. A. (2019). An Overview of The Traumatic Brain Injury-Quality of Life (TBI-QOL) Measurement System. J. Head Trauma Rehabil. 34, 281–288. doi: 10.1097/HTR.0000000000000531, PMID: 31498227

[ref62] Tulsky D. S. Kisala P. A. Kalpakjian C. Z. Bombardier C. H. Pohlig R. T. Heinemann A. W. (2015a). Measuring depression after Spinal Cord Injury: Development and Psychometric Characteristics of the SCI-QOL Depression Item Bank And Linkage With PHQ-9. J. Spinal Cord Med. 38, 335–346. doi: 10.1179/2045772315Y.0000000020, PMID: 26010968 PMC4445024

[ref63] Tulsky D Kisala P Tyner C Slotkin J Kaufman C Dearth C (2023). Identifying health-related quality of life domains after upper extremity transplantation. Arch. Phys. Med. Rehabil. doi: 10.1016/j.apmr.2023.01.001 36639091

[ref64] Tulsky D. S. Kisala P. A. Victorson D. Carlozzi N. Bushnik T. Sherer M. (2016). TBI-QOL: Development and Calibration of Item Banks to Measure Patient Reported Outcomes Following Traumatic Brain Injury. J. Head Trauma Rehabil. 31, 40–51. doi: 10.1097/HTR.0000000000000131, PMID: 25931184 PMC4697960

[ref65] Tulsky D. S. Kisala P. A. Victorson D. Choi S. W. Gershon R. Heinemann A. W. (2015b). Methodology for the development and calibration of the SCI-QOL Item Banks. J. Spinal Cord Med. 38, 270–287. doi: 10.1179/2045772315Y.0000000034, PMID: 26010963 PMC4445019

[ref66] Tulsky D. S. Kisala P. A. Victorson D. Tate D. Heinemann A. W. Amtmann D. (2011). Developing a contemporary patient-reported outcomes measure for spinal cord injury. Arch. Phys. Med. Rehabil. 92, S44–S51. doi: 10.1016/j.apmr.2011.04.024, PMID: 21958922 PMC6309317

[ref67] Tulsky D. S. Kisala P. A. Victorson D. Tate D. G. Heinemann A. W. Charlifue S. (2015c). Overview of the Spinal Cord Injury--Quality of Life (SCI-QOL) Measurement System. J. Spinal Cord Med. 38, 257–269. doi: 10.1179/2045772315Y.0000000023, PMID: 26010962 PMC4445018

[ref68] Tyner C Wyatt M Pruziner A Cancio J Slotkin J Kisala P (2018). “Quality-of-life assessment after major extremity trauma: developing a new patient-reported outcome measure.” in *Oral Presentation at the Military Health System Research Symposium (MHSRS)*; Kissimmee, FL.

[ref69] Victorson D. Tulsky D. S. Kisala P. A. Kalpakjian C. Z. Weiland B. Choi S. W. (2015). Measuring resilience after Spinal Cord Injury: Development, Validation and Psychometric Characteristics of the SCI-QOL Resilience Item Bank And Short Form. J. Spinal Cord Med. 38, 366–376. doi: 10.1179/2045772315Y.0000000016, PMID: 26010971 PMC4445027

